# Relation of Ultrasound Findings and Abdominal Symptoms obtained with the CFAbd-Score in Cystic Fibrosis Patients

**DOI:** 10.1038/s41598-017-17302-4

**Published:** 2017-12-12

**Authors:** Harold Tabori, Anke Jaudszus, Christin Arnold, Hans-Joachim Mentzel, Michael Lorenz, Ruth K. Michl, Thomas Lehmann, Diane M. Renz, Jochen G. Mainz

**Affiliations:** 1 Jena University Hospital, Cystic Fibrosis Centre, Jena, Germany; 2 Jena University Hospital, Pediatric Radiology, Jena, Germany; 3 Jena University Hospital, Department of Internal Medicine IV (Gastroenterology, Hepatology, and Infectious Diseases), Jena, Germany; 4 Jena University Hospital, Institute of Medical Statistics, Jena, Germany

## Abstract

Abdominal symptoms are a hallmark of Cystic fibrosis (CF). Yet, their association with morphological abnormalities of different abdominal organs is still poorly understood. Aim was therefore to relate these symptoms, assessed with a questionnaire, to findings in abdominal ultrasound (US). In 114 CF patients of all ages, findings in US considering seventeen specific parameters were related to abdominal symptoms compiled with our novel CF-specific 26-modal symptom score (CFAbd-Score). US abnormalities were detected in 95% of the patients. Most frequent findings were pancreatic lipomatosis (88%), liver steatosis (37%), hepatomegaly (31%), and thickened bowel walls (23%). Highest burden of GI-symptoms was clearly associated with pancreatic lipomatosis (p = 0.036). In detail, patients revealing this pathology reported higher rates of abdominal pain (p = 0.018), flatulence (p = 0.006), heartburn (p = 0.04), and reflux of stomach content (p = 0.006). Patients with pancreatic sufficiency had less US-findings (p = 0.033), which in turn was associated with lower rates of abdominal symptoms. The majority of them were carriers of class IV-VI or G551D mutations. Our approach gives new insights regarding the underestimated multi-organ abdominal involvement in CF. The new score can be of high interest e.g. as a complementary tool to assess the gastrointestinal effects of promising novel CF therapeutics.

## Introduction

Whereas pulmonary involvement in cystic fibrosis (CF), the most frequent autosomal recessive lethal disorder in Caucasians, has been intensively studied, the abdominal manifestations in CF are still not sufficiently understood. Abdominal manifestations, which substantially contribute to the high burden of symptoms and to preliminary death in CF, include pancreatic insufficiency (PI), focal biliary cirrhosis, micro-gallbladder with sludge and concrements, meconium ileus (MI), intestinal prolapses and intussusception and, in higher ages, distal intestinal obstruction syndrome (DIOS)^1^. Dysfunction of the cystic fibrosis transmembrane conductance regulator (*CFTR*) in the pancreatic and biliary ducts and in intestinal epithelia results in viscous acidic secretions leading to lumen obstruction and impaired digestion. The resulting deficiency of nutrients and fat-soluble vitamins, failure to thrive and reduced body weight are strongly correlated to impaired pulmonary function and reduced survival^2^. Ultrasound (US) has a high value for detecting abdominal pathologies non-invasively and without exposition to radiation. In our center, US has been established as routine control, performed every 6 to 12 months in CF patients. It allows static and dynamic assessment of pathologies such as pancreatic lipomatosis and cystosis, liver abnormalities, and bowel wall thickness^3^.

**Figure 1:**
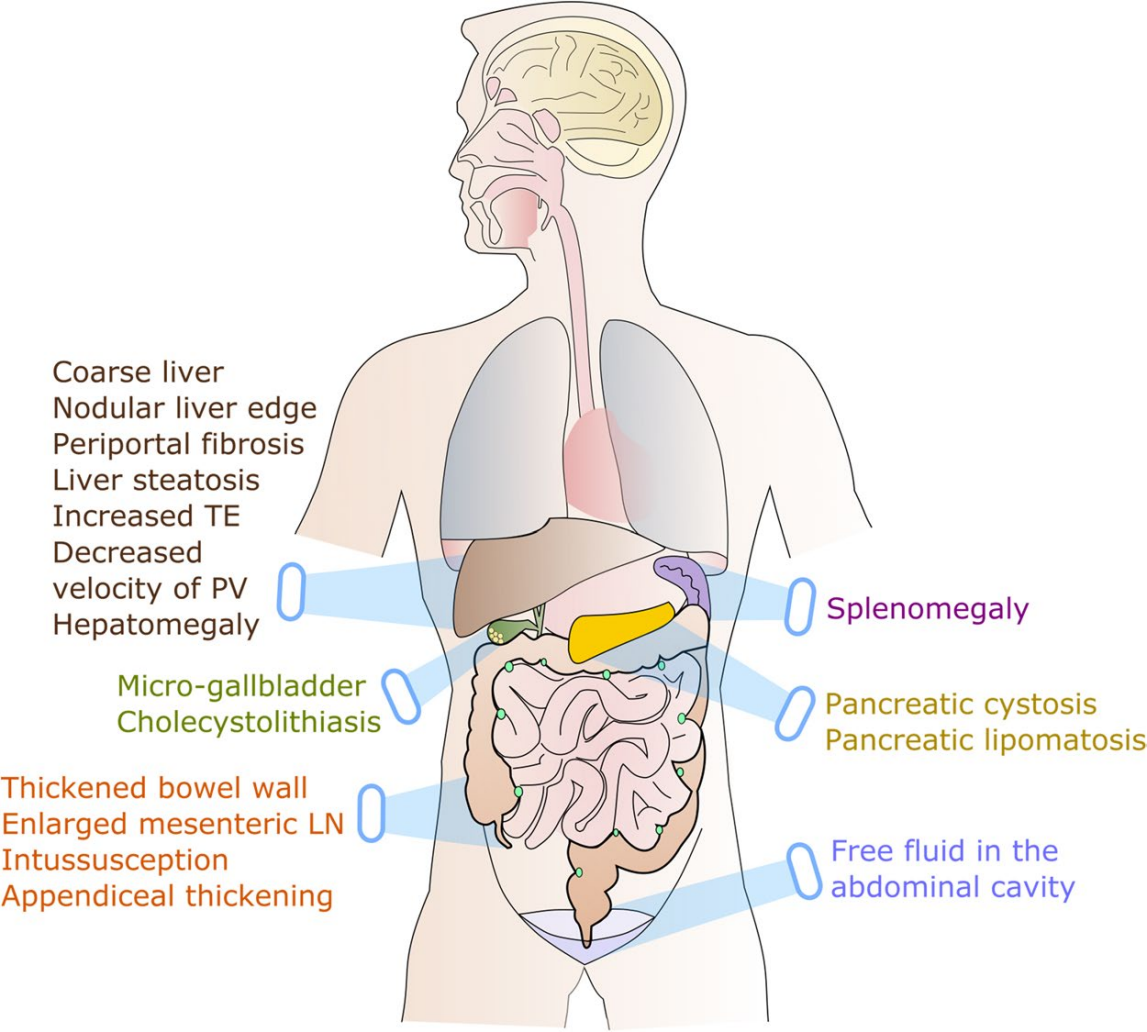
Seventeen abdominal abnormalities in cystic fibrosis detected with ultrasound. TE: transient elastography; PV: (maximal flow velocity in) portal vein; LN: lymph nodes.

With our recently presented JenAbdomen-Score we showed that, among the complex symptoms, abdominal pain and distention, flatulence, lack of appetite and nausea, fatty stools, and diarrhea are most prominent^4^. Yet, the relation of these symptoms to morphological abnormalities of different abdominal organs is still poorly understood. Therefore, we recorded and quantified the complex GI symptoms with a revised, improved, and now 26-modal version of our questionnaire (CFAbd-Score) and related the calculated scores to morphological findings obtained with structured abdominal US, assessing seventeen parameters which are frequently abnormal in CF (Fig. 1).

## Materials and Methods

### Participants and Settings

The prospective study was performed consecutively including CF patients of all ages attended at the Jena University Hospital CF Center. Inclusion criteria were: (1) a diagnosis of CF determined by a sweat chloride of >60 mEq/L and/or (2) detection of 2 disease causing *CFTR* mutations with evidence of organ involvement. We considered patients of all ages who completed the self-reported or parent-proxy reported CFAbd-Score.

### Ethical Statement

The study was approved by the Jena University ethics committee (registration number 4458-06/15) and done in adherence to ethical principles. All methods were performed in accordance with the relevant guidelines and regulations. All patients and parents of minors provided written informed consent.

### Evaluation of abdominal symptoms

The CFAbd-Score was further developed from our recently presented JenaAbdomen-CF Score 1.0^4^ with additional 9 items concerning GI symptoms-related quality of life (QoL), thus now consisting of 26 items. QoL items were: embarrassed, physical activity limitation, reduced productivity, fatigue, reduced concentration, frustrated/restless/irritable, sad, difficulty falling asleep, and waking up at night, measured with a 6-point Likert scale from ‘not at all’ (0 pts) to ‘always’ (5 pts).

In contrast to the first version, each item was reversely scored and linearly transformed to a 100-points scale (0 = 100, 1 = 80, 2 = 60, 3 = 40, 4 = 20, 5 = 0) with lower rates for increasing severity of GI symptoms. Only the domains with more than 50% of the items answered were included in the calculation. CFAbd-Score (26 items) was calculated as the sum of the items over the number of items answered in each domain.

### Measurement of ultrasound abnormalities

The US examinations were performed in a single center using an ultrasound scanner (Philips; U22; Philips Healthcare, Best, the Netherlands) under standardized conditions. The findings were confirmed by and discussed with a second experienced radiologist in order to enhance the quality of the data. The abdominal US findings included evaluation of 17 parameters:

1. *Bowel wall thickness* (BWT) was measured in a longitudinal and transverse section. The measurement was taken from the central hyperechoic line of the lumen (representing the interface between content of the lumen and the mucosa) to the outer hyperechoic margin of the wall (representing the serosa). BWT was considered ‘normal’ up to 4 mm^5^.
2. *Enlarged mesenteric lymph nodes* were defined as greater than 5 mm in the shorter axis and larger than 10 mm in the long axis^5^.
3. *Intussusception* was defined as the invagination of a proximal segment of the intestine into an adjacent distal segment. In US, it appears as a mass with multiple concentric rings or doughnut signs on the short axis^5^.
4. *Appendiceal thickening* was defined as a diameter of more than 6 mm and/or an appendiceal wall thicker than 2 mm^6^.
5. *Free fluid* within the peritoneal cavity.
6. *Pancreatic cystosis* was defined as anechoic structures which are usually round and oval (cystic lesions) with sizes greater than 1 cm (macroscopic cysts)^7^.
7. *Pancreatic lipomatosis* was given when pancreatic echogenicity was *partially* or *completely* higher than liver (or the kidney in case of liver hyperechogenity)^8^.
8. * Cholecystolithiasis* was demonstrated by typical acoustic shadow in the gallbladder.
9. *Micro-gallbladder* was defined as less than 2–3 cm long and 0.5–1.5 cm wide^9^.
10. *Coarseness of the hepatic parenchyma*,
11. *nodularity of the liver edge*, and
12. *periportal fibrosis* (increased periportal echoes) were documented according to a scoring system established by Williams *et al*.^10^.
13. *Liver steatosis* criteria were increased echogenicity compared to renal parenchyma, vascular blurring, and deep attenuation of the US signal^11^.
14. The *maximum velocity of flow in the portal vein* (PV) was measured in cm/s and was considered decreased below 15 cm/s^12^.
15. *Transient elastography* (TE) was measured in kPa and was considered increased with a value above 7.1 kPa^13^.
16. *Hepatomegaly* was indicated when the liver span at the mid-clavicular line exceeded the upper limits according to height^14^.
17. *Splenomegaly* was indicated when spleen length (measured as the larger diameter through the hilum in a cranio-caudal axis) exceeded the upper limits according to age^15^.

These 17 parameters were scored either as absent (0 pts) or present (1 pt) with a maximal score of 17 points (US-17). In addition to US-17, the Williams score^10^ and TE were correlated separately to the CFAbd-Score.

In order to link the pancreatic status (pancreas sufficiency = PS/pancreas insufficiency = PI) to US abnormalities, doses of substituted enzymes quantified as intake of international units of pancreatic lipase per kg of body weight and day (IU/kg/d) were additionally factored in.

### Measures of clinical data

The recently established pancreatic insufficiency prevalence (PIP) scores adapted from Ooi were used to measure the severity of specific *CFTR* mutations in regard to pancreatic function^16^. Patients carrying mutations which had not been included into the study from Ooi could not be attributed to a specific PIP score and thus were excluded from the PIP-genotype analysis (12/114 patients). *CFTR-*mutations were classified as I-III (severe) and IV-V (mild)^17^. An adequate visualization of the pancreas by US was present in 92% of the patients (105/114). TE could be performed in 99 of the 114 patients. CF-liver disease (CFLD) was defined according to Debray *et al*.^18^.

### Data Analysis

Statistical analyses were performed using SPSS v.23.0. Median, lower (Q_1_) and upper (Q_3_) interquartiles of the score in relation to the respective US parameter are given as median [Q_1_;Q_3_]. To detect statistical differences between the US findings-associated symptom scores, Mann-Whitney-U test was chosen following Kolmogorov-Smirnov (K-S) testing of normal distribution. To evaluate the difference between two patient groups (e.g. PS vs. PI), the Hodges-Lehmann (HL) estimator (median of all pairwise differences) with 95% confidence interval (CI) was reported. Nominal data was compared with the Chi-square test or Fisher’s exact tests, as appropriate. Correlations between variables were examined using the Pearson’s correlation coefficient. A p-value ≤ 0.05 indicates a significant difference or correlation.

## Results

One-hundred fourteen patients (52.6% females) were enrolled prospectively. The mean age was 19.8 ± 13.6 (1–75) years. Exocrine PI was present in 106 patients (93%, Table 1) at time of inclusion, 8 patients were PS and 9 patients (8%) had undergone bowel resection. *CFTR* mutations were identified on both alleles in all patients.

**Table 1:** Characteristics of the included CF patients (n = 114).

	Variable	N (%)
Sex	Female	61 (52.6%)
	Male	53 (47.4%)
Genotype	*F508del*/*F508del*	48 (42.1%)
	*F508del*/*other*	53 (46.5%)
	*G551D*/*other*	15 (13.0%)
	*Other*/*other*	13 (10.7%)
Age (yrs.)	0–5	17 (14.9%)
	6–11	25 (21.9%)
	12–17	14 (12.3%)
	≥18	58 (50.9%)
Therapy	Antibiotic therapy in the previous 3 months	84 (73.7%)
	Pancreatic enzymes intake	107 (93.9%)
	Proton pump inhibitors (PPI)	30 (36.3%)
Comorbidities	Exocrine pancreatic insufficiency (EPI)	106 (93.0%)
	Meconium ileus (MI)	9 (7.9%)
	Distal intestinal obstruction syndrome (DIOS)	11 (9.6%)
	Rectal prolapse	13 (11.4%)
	CF liver disease (CFLD)	21 (18.4%)
	Small bowel resection	9 (7.9%)
Serum	Elevated^†^ ALT	60 (52.6%)
	Elevated^†^ AST	25 (21.9%)
	Elevated^†^ γ-GT	12 (10.5%)
	Elevated^†^ AP	48 (42.1%)
	Platelet counts reduced	5 (4.4%)

^†^Elevated in regard to age and gender-related reference.

ALT: alanine aminotransferase; AST: aspartate aminotransferase; γ-GT: γ-glutamyl transpeptidase;

and AP: alkaline phosphatase.

### Relation of US-findings to symptoms assessed with the CFAbd-Score

**Figure 2:**
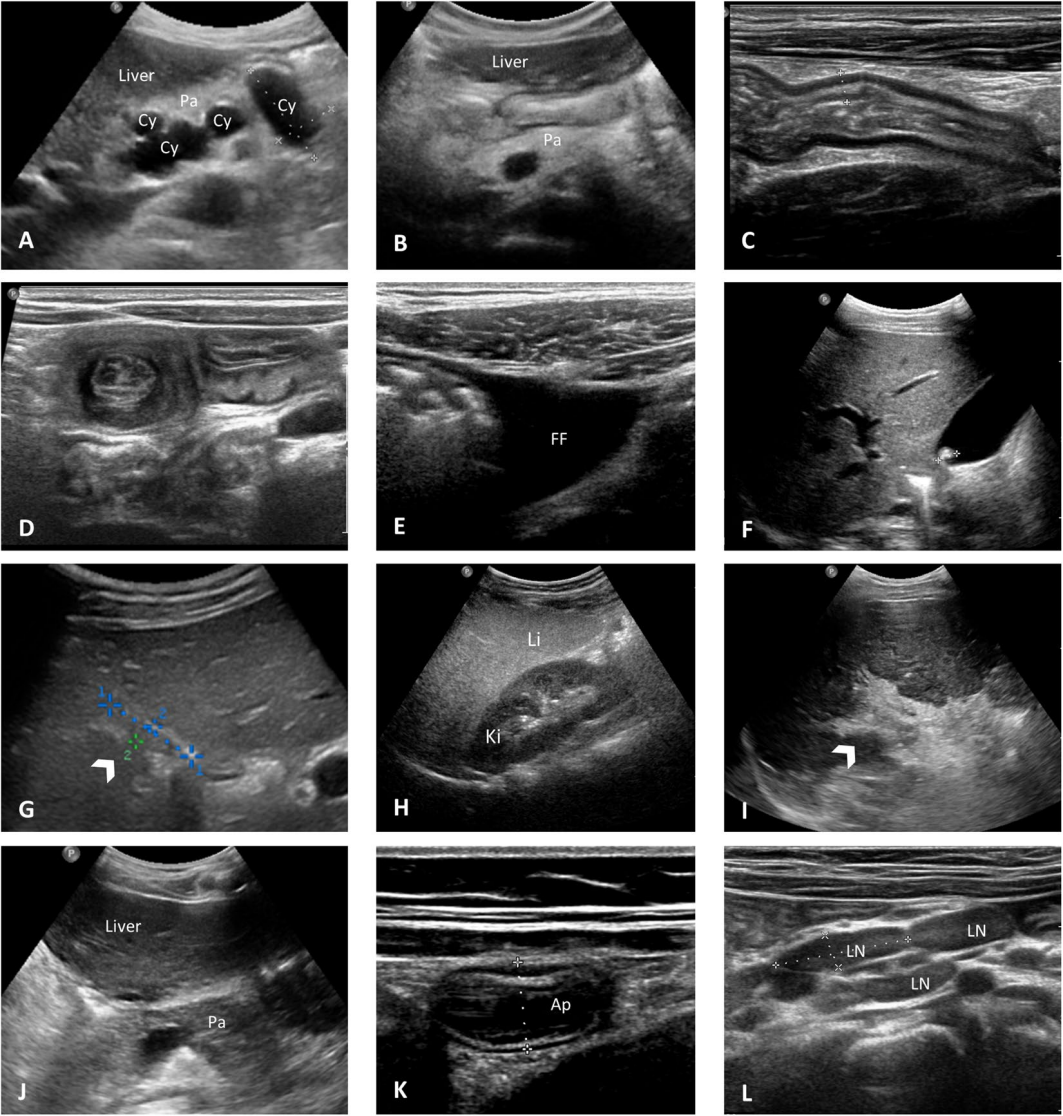
Specific US abnormalities identified in the Jena University CF cohort. (**A**) Pancreatic cystosis in an asymptomatic 41-year-old female patient heterozygous for *G551D* showing a hyperechoic pancreas (Pa) containing multiple cysts (Cy) with sizes approximately of 21 × 34 × 20 mm and 21 × 40 × 24 mm. CFAbd-Score: 82 (*range: highest burden of symptoms [0 points] to no symptoms [100 points], respectively). (**B**) Hyperechoic pancreas (Pa) with fat replacement as typical for pancreatic lipomatosis in a 10-year-old female patient homozygous for *F508del* with pancreatic insufficiency (symptom score: 95). (**C**) Longitudinal US image of the bowel wall in the terminal ileum measuring approximately 5 mm in a context of thickened bowel wall in a 19-year-old patient homozygous for *F508del* (symptom score: 81). (**D**) Transversal section of the small bowel showing the classic target sign appearance of intussusception in a 3-year-old female asymptomatic patient homozygous for *F508del* (symptom score: 96). (**E**) Free fluid (FF) in the right lower quadrant in a 32-year-old male patient homozygous for *F508del* with severe liver disease (symptom score: 80). (**F**) Single gallstone in a 12-year-old male asymptomatic patient heterozygous for *G551D* (symptom score: 100). (**G**) Gallbladder (arrow) in a 11-year-old female patient heterozygous for *G551D* measuring 18 × 4 mm in a context of micro-gallbladder (symptom score: 88). (**H**) US image of liver steatosis in a 4-year-old male patient homozygous for *F508del*, showing diffused increased echogenicity of the right lobe of the liver (Li) relative to right kidney cortex (Ki) (symptom score: 85). I) Severe periportal fibrosis (arrow) in a 28-year-old male patient heterozygous for *F508del/R347P w*ith cystic fibrosis liver disease (CFLD) (symptom score: 87). (**J**) Isoechoic pancreas (Pa) relative to liver in a 18 year-old female patient heterozygous for *G551D* with pancreatic sufficiency (PS) in longitudinal sonogram at level of the pancreatic head (symptom score: 99). (**K**) US scan of the appendix (Ap) in a 10-year-old asymptomatic female patient homozygous for *F508del*. Note the thickened aspect of the appendix (diameter of 9 mm) (symptom score: 95). (**L**) Enlarged mesenteric lymph nodes (LN) greater than 18 mm in the larger axis in a 10-year-old asymptomatic patient homozygous for *F508del*, in a context of an inflammatory etiology (symptom score: 95).

Ultrasound abnormalities detected in the included CF patients are listed in Table 2 and Fig. 2. Altogether, patients with pancreatic lipomatosis detected by abdominal US revealed lower values for CFAbd-Score (84 [76;91] vs. 94 [81;95]; p = 0.036) according to a higher burden of symptoms than those without pancreatic lipomatosis. In detail, they reported a higher burden of abdominal pain (AP) frequency (HL = 20 pts, 95% CI [0;20]; MWW p = 0.018), AP duration (HL = 0 pts, 95% CI [0;20]; MWW p = 0.046), and AP intensity (HL = 20 pts, 95% CI [10;30]; MWW p = 0.002) as well as flatulence (HL = 20 pts, 95% CI [0;20]; MWW p = 0.006), heartburn (HL = 0 pts, 95% CI [0;20]; MWW p = 0.04), and reflux of stomach content (HL = 0 pts, 95% CI [0;20]; MWW p = 0.006) (Supplementary Table S1). Furthermore, patients with micro-gallbladder detected in US reported higher rates of pain during bowel movements (HL = 10 pts, 95% CI [0;10]; MWW p = 0.014) whereas patients with liver steatosis more frequently suffered from fatty stools (HL = 0 pts, 95% CI [0;20]; MWW p = 0.031). At the same time, we did not detect significant differences in the CFAbd-Score with thickened bowel walls (n = 26/114), enlarged mesenteric lymph nodes (n = 10/114), appendiceal thickening (n = 7/114), free abdominal fluid (n = 11/114), pancreatic cystosis (n = 6/105), hepatic parenchymal abnormalities such as coarse/irregular parenchyma (n = 25/114), nodular liver edge (n = 7/114), periportal fibrosis (n = 19/114), as well as hepatomegaly (n = 35/114), splenomegaly (n = 12/114), decreased velocity of PV (n = 11/114) and increased TE (n = 7/99). Cholecystolithiasis (n = 3/114) and intussusception (n = 2/114) were not evaluated because of the small number of patients with these pathologies detected in US. In general, CFAbd-Score did not correlate with US-17 scores (r = 0.01; p = 0.92), maximum velocity of PV (r = 0.17; p = 0.08), TE values (r = 0.17; p = 0.09), or the Williams score (r = 0.02; p = 0.86).

**Table 2:** Frequencies of detected abnormalities in abdominal ultrasound (US-17).

Pathologies detected by abdominal US	N (%)
Pancreatic lipomatosis^†^	92/105 (88)
Liver steatosis	42/114 (37)
Hepatomegaly	35/114 (31)
Thickened bowel wall (>4 mm)	26/114 (23)
Coarse/irregular hepatic parenchyma	25/114 (22)
Micro-gallbladder	24/114 (21)
Periportal fibrosis	19/114 (17)
Splenomegaly	12/114 (11)
Free fluid in the abdominal cavity	11/114 (10)
Decreased velocity of PV	11/114 (10)
Enlarged mesenteric lymph nodes	10/114 (9)
Increased TE*	7/ 99 (7)
Appendiceal thickening	7/114 (6)
Nodular liver edge	7/114 (6)
Pancreatic cystosis^†^	6/105 (6)
Cholelithiasis	3/114 (3)
Intussusception	2/114 (2)

^†^Pancreas was adequately visualized in 105 of the 114 patients.

*Transient elastography (TE) was performed in 99 of the 114 patients.

Furthermore, we did not find differences in US-17 between sexes. Altogether, only 5.3% of the included CF patients (6/114) did not reveal any pathological US-finding, which in part also could have been caused by superposition of the pancreas or other organs with bowel gases (minimal abdominal US). Moreover, enzyme dose was not related to US abnormalities (Supplementary Table S2).

### US-findings in relation to the pancreatic status

Comparison of ultrasound findings between PS and PI patients revealed pancreatic lipomatosis in 75% vs. 91%, (p = 0.034) and liver steatosis in 13% vs. 42% of patients, respectively (p = 0.021) (Supplementary Table S3). Accordingly, pancreatic sufficient patients showed an overall lower frequency of US-findings compared with patients with PI (1.5 [0;2] vs. 3 [2;4]; p = 0.033).

### Correlations between *CFTR* genotype and ultrasound findings (US-17)

The number of US findings (US-17) showed a weak positive correlation with the PIP-score (r = 0.26; p < 0.01). Patients carrying at least one class IV to VI mutation revealed lower rates of pathologies in US-17 than patients carrying class I to III mutations (1 [1;2] vs. 3 [2;4]; p = 0.004). Thereby, patients carrying a *G551D* mutation (≈13% of the CF patients attended in the Jena University CF center) showed a lower number of US findings (US-Score) compared to those without this mutation (2 [1;3] vs. 3 [2;5]; p = 0.018). However, 8/15 of these patients received ivacaftor at the time of inclusion, so that we cannot determine whether this outcome is primary for patients with a *G551D* mutation or secondary due to therapy with the potent *CFTR* modulator.

## Discussion

To our knowledge, this is the first study correlating the wide range of different findings in abdominal ultrasound in CF patients with the range of typical abdominal symptoms assessed with a second, revised version of our CF-specific score (CFAbd-Score). Thereby, we queried frequency, duration, and intensity of symptoms together with the related burden for the patient’s daily life. By using US measures we found that particularly pancreatic lipomatosis is associated with an increased abdominal symptom load. Altogether, pancreatic lipomatosis was detected in 88% (92/105) of the included CF patients. Overall, this accords well to the pancreatic status, as the majority of the included CF patients with pancreatic lipomatosis were pancreas insufficient (PI) (91%), and these US changes are a common finding in this subgroup^13,19^. By means of serial radiology, four states have been revealed in the course of the progression from PS to PI: normal pancreas, an atrophic state subsequent to recurrent pancreatitis, a lipomatous state in which pancreatic tissue is successively replaced by fat-dense tissue, and a macro- or microcystic pancreas with parietal calcifications^20^. Finally, many patients who initially were classified as PS will become PI. The finding of lipomatosis in our PS patients indicates an advanced stage in the progression from PS to PI. On the basis of a first and preliminary version of our score (JenAbdomen-CF Score 1.0) we demonstrated that PI-CF patients report significantly more GI symptoms compared to PS-CF patients^4^. A recent study using a symptom questionnaire developed as PI-specific patient-reported outcome measure (PROM) revealed that 84% of the patients reported experiencing abdominal pain^21^. This is quite similar to our finding that 79% of our CF patients (92% PI) recurrently and regularly suffer from abdominal pain. The most frequent locations of abdominal pain were the umbilical (83%) and epigastric regions (11%)^4^. Our finding on the impact of a pancreatic lipomatosis fits very well with the patients´ statements. The elevated rate of GI symptoms in PI patients may be caused by the combination of exocrine pancreatic deficiency to liberate enzymes, the failure to neutralize the acidic gastric contents, and intestinal inflammation^22^. Nevertheless, this finding should be taken carefully, as a smaller number of patients with elevated pancreatic echogenicity quoted as pancreatic lipomatosis still maintain residual pancreatic function according to a PS phenotype and vice versa. At the same time, US revealing a hypoechoic pancreas, which does not accord to pancreatic lipomatosis, could represent a fibrotic pancreas, as found in patients with PI^19^. However, these cases accounted for a minority of our cohort.

Interestingly, prominent US findings such as pancreatic cystosis, which was detected in 6/105 patients (6%), with cyst lesions measuring up to 40 mm did not significantly contribute to the burden of abdominal symptoms in these patients. However, this specific pathology might cause symptoms later, e.g. when further growth of cysts compress surrounding structures, as described in a single case report by deGruchy *et al*.: the 9 year old patient with pancreatic cystosis suffered from intense radiating abdominal pain^23^. Previously, Dietrich *et al*. reported that abdominal US reveals small pancreatic cysts with a mean diameter of 18 mm (7–27 mm) in up to 18% of CF patients (n = 12/67)^9^.

Thickened bowel walls, as detected in 26 of 114 patients (23%) of our CF cohort, were not significantly correlated with an increased burden of GI symptoms. Thereby, until now, etiology of bowel wall thickening in CF is not fully understood. A relation to intestinal inflammation, and possibly to dysbiosis has been discussed and also that in the long run it could lead to segmental submucosal fibrosis^24^. However, this assumption needs further evaluation in future studies. As previously reported, the dose of applied pancreatic enzymes did not correlate with the presence of thickened bowel walls^24^, even though such a correlation was discussed^25^.

The presence of an enlarged appendix was observed in 7 of the included CF patients (6%). This finding was not related to an increased burden of GI symptoms. Apparently, patients remain asymptomatic until occurrence of an appendiceal perforation or abscess formation^6^. Altogether, the appendix in CF has been reported to be usually filled with mucoid contents, which maintain the appendiceal lumen distended and less prone to total luminal occlusion and acute inflammation. Consequently, progress to acute appendicitis in CF appears to be rare, occurring in only 1–2% of patients^26^.

As suspected, hepatic abnormalities including coarse parenchyma, nodular edges, periportal fibrosis, organomegaly, increased TE, and decreased PV velocity was not correlated with an increased burden of GI symptoms. However, symptoms like upper GI hemorrhage and tarry stools may manifest with end-stage liver disease^27^. Such an acute hepatic decompensation, which is often associated with metabolic disorders, is rare and did not occur in our observational period.

In the present study, 42 patients (37%) revealed sonographic signs of liver steatosis, which is in accordance with previous ultrasound studies reporting steatosis in 23–75% of CF patients^28^. The most common abdominal symptom found in that subgroup was ‘fatty stools´, which was significantly more frequent in this group (p = 0.031). Interestingly, 41 out of these 42 patients were PI. As recently published, patients who underwent pancreatectomy are likely to develop hepatosteatosis due to maldigestion. This largely depended on the endo- and exocrine function of the remnant pancreas and a well-adjusted enzyme supplementation^29^. In non-CF patients, hepatosteatosis is closely linked to an impaired glucose metabolism^30^. It is conceivable that also in CF the development of a liver steatosis is promoted by insulin deficiency due to pancreatic insufficiency. Once in progress, the situation is worsened by an inadequate synthesis of hepatic phosphatidylcholine and apolipoprotein B, which results in impaired VLDL assembly and, subsequently, disturbed removal of triacylgycerols from the liver^1,31,32^. Moreover, deficiency of essential fatty acids and carnitine are factors discussed in the context of hepatosteatosis in CF^33^.

Enlarged abdominal lymph nodes, consistently with a reactive morphology, were detected in 10 of our patients (9%). This finding was not associated with elevated GI symptomatology. Likewise, detection of gallstones (found in 3% of patients) was not related to elevated symptoms. However, it is not unlikely to give rise to symptoms in the course of time.

Interestingly, PS patients carrying a mild genotype (class IV-VI) as well as patients with a *G551D* mutation had significantly lower rates of US abnormalities (US-17) compared to patients with class I-II mutations and PI, respectively. As a limitation, 8 of the 15 patients carrying a *G551D* mutation were treated with ivacaftor (IVA) at the time of inclusion. With higher patient numbers, it would be most interesting to assess changes of abdominal symptoms due to introduction of the potent *CFTR* modulator. According to recent publications, IVA improved many of the abdominal pathologies such as a hyperacidic gastric content and liver steatosis, and even restored exocrine pancreatic function to some extent in some patients^34–36^. Even more impressive, improvement of GI symptoms was observed in CF patients treated with orkambi, the combination of the *CFTR* corrector lumacaftor and the potentiator IVA, which was recently approved for CF patients homozygous for *F508del* in many countries. Here is where a differential score assessing the multimodal abdominal involvement in CF is of outstanding interest to sufficiently document and quantify such interesting effects. This would be one of many interesting fields for the application of a score like the present CFAbd-Score, which could bring substantial new insights as a PROM. As previously mentioned, the validation of this PROM is still in progress and, just like the JenAbdomen-CF Score 1.0, the second version presented herein is preliminary^4^. Nevertheless, we show that especially pancreatic lipomatosis was well reflected by self-reported elevated GI-symptoms. Our data may contribute to the still little understanding of the abdominal involvement in CF.

## Electronic supplementary material

Tables S1-3
