# Supplementary material for: Relation of Ultrasound Findings and Abdominal Symptoms obtained with the CFAbd-Score in Cystic Fibrosis Patients
Source: Sci Rep. 2017 Dec 12;7:17465. doi: 10.1038/s41598-017-17302-4 (PMC5727223; doi:10.1038/s41598-017-17302-4)
Supplement: Supplementary file 1 — Tables S1-3 [file 41598_2017_17302_MOESM1_ESM.doc]

**Relation of Ultrasound Findings and Abdominal Symptoms obtained with the CFAbd-Score in Cystic Fibrosis Patients**

Harold Tabori, Anke Jaudszus, Christin Arnold, Hans-Joachim Mentzel, Michael Lorenz, Ruth K. Michl, Thomas Lehmann, Diane M. Renz, Jochen G. Mainz

**Table S1:** Medians of symptoms scores quantified with the CFAbd-Score in relation with the ultrasound findings^§^

|  |  |  | **CFAbd-Score** | | | | | | | | | | | |
| --- | --- | --- | --- | --- | --- | --- | --- | --- | --- | --- | --- | --- | --- | --- |
|  |  |  | **AP-4** | | | | **NAP-8** | | | | | | | |
| **US-findings** |  | N | APF | APD | API | APB | Fla | AD | FS | Cnst | HB | Vom | Nau | Ref |
| **Thickened bowel wall** | Yes | 26 | 80 [60;80] | 80 [70;80] | 80 [70;80] | 100 [90;100] | 80 [40;80] | 80 [75;85] | 80 [60;80] | 80 [80;100] | 90 [60;100] | 100 [100;100] | 100 [80;100] | 90 [80;100] |
|  | No | 88 | 80 [60;80] | 80 [65;80] | 70 [60;90] | 100 [80;100] | 80 [60;80] | 80 [60;100] | 80 [60;80] | 100 [80;100] | 100 [80;100] | 100 [80;100] | 100 [80;100] | 100 [80;100] |
| **Enlarged lymph nodes** | Yes | 10 | 80 [60;80] | 80 [65;80] | 70 [45;85] | 100 [68;100] | 80 [75;85] | 100 [70;100] | 70 [60;100] | 100 [75;100] | 100 [100;100] | 90 [80;100] | 80 [80;100] | 100 [80;100] |
|  | No | 104 | 80 [60;80] | 80 [70;80] | 80 [60;80] | 100 [90;100] | 80 [60;80] | 80 [60;100] | 80 [60;80] | 100 [80;100] | 100 [80;100] | 100 [100;100] | 100 [80;100] | 100 [80;100] |
| **Appendiceal thickening** | Yes | 7 | 80 [80;80] | 80 [80;100] | 70 [60;90] | 100 [98;100] | 80 [80;100] | 100 [60;100] | 70 [60;100] | 100 [80;100] | 100 [100;100] | 100 [80;100] | 100 [80;100] | 100 [100;100] |
|  | No | 107 | 80 [60;80] | 80 [60;80] | 70 [60;80] | 100 [80;100] | 80 [60;80] | 80 [60;100] | 80 [60;80] | 100 [80;100] | 100 [80;100] | 100 [100;100] | 100 [80;100] | 100 [80;100] |
| **Free fluid** | Yes | 11 | 80 [60;80] | 80 [80;80] | 70 [70;80] | 100 [98;100] | 80 [80;100] | 90 [75;100] | 80 [60;80] | 80 [80;100] | 100 [100;100] | 100 [100;100] | 100 [80;100] | 100 [100;100] |
|  | No | 103 | 80 [60;80] | 80 [60;80] | 80 [60;80] | 100 [80;100] | 80 [60;80] | 80 [60;100] | 80 [60;80] | 100 [80;100] | 100 [80;100] | 100 [80;100] | 100 [80;100] | 100 [80;100] |
| **Pancreatic cystosis** | Yes | 6 | 80 [60;85] | 80 [80;90] | 85 [68;100] | 90 [78;100] | 80 [55;85] | 80 [75;85] | 70 [55;85] | 100 [80;100] | 90 [75;100] | 100 [100;100] | 100 [95;100] | 100 [95;100] |
|  | No | 99 | 80 [60;80] | 80 [80;80] | 80 [60;80] | 100 [90;100] | 80 [60;80] | 80 [60;100] | 80 [60;80] | 100 [80;100] | 100 [80;100] | 100 [80;100] | 100 [80;100] | 100 [80;100] |
| **Pancreatic lipomatosis** | Yes | 92 | **80 [60;80]*** | **80 [75;80]*** | **70 [60;80]*** | 100 [90;100] | **80 [60;80]*** | 80 [60;100] | 80 [60;70] | 100 [80;100] | **100 [80;100]*** | 100 [100;100] | 100 [80;100] | **100 [80;100]*** |
|  | No | 13 | **80 [80;100]** | **80 [80;100]** | **95 [80;100]** | 100 [83;100] | **80 [80;100]** | 100 [80;100] | 80 [80;100] | 100 [80;100] | **100 [100;100]** | 100 [80;100] | 100 [90;100] | **100 [100;100]** |
| **Micro-gallbladder** | Yes | 24 | 80 [60;80] | 80 [60;80] | 70 [50;80] | **90 [80;100]*** | 80 [60;80] | 80 [60;85] | 80 [60;80] | 100 [80;100] | 100 [80;100] | 100 [80;100] | 800 [80;100] | 80 [80;100] |
|  | No | 90 | 80 [60;80] | 80 [80;80] | 70 [60;80] | **100 [90;100]** | 80 [60;80] | 80 [80;100] | 80 [60;80] | 100 [80;100] | 100 [80;100] | 100 [95;100] | 100 [80;100] | 100 [80;100] |
| **Coarse/Irregular hepatic parenchyma** | Yes | 25 | 80 [60;80] | 80 [65;80] | 70 [50;80] | 100 [90;100] | 80 [60;80] | 80 [80;100] | 60 [60;95] | 100 [80;100] | 90 [65;100] | 100 [90;100] | 100 [80;100] | 100 [80;100] |
|  | No | 89 | 80 [60;80] | 80 [70;80] | 80 [60;80] | 100 [80;100] | 80 [60;80] | 80 [60;100] | 80 [60;80] | 100 [80;100] | 100 [80;100] | 100 [80;100] | 100 [80;100] | 100 [80;100] |
| **Nodular liver edge** | Yes | 7 | 80 [40;80] | 80 [80;100] | 70 [50;80] | 100 [100;100] | 80 [60;100] | 80 [80;100] | 90 [45;100] | 100 [80;100] | 100 [100;100] | 100 [80;100] | 80 [80;100] | 100 [100;100] |
|  | No | 107 | 80 [60;80] | 80 [60;80] | 70 [60;80] | 100 [83;100] | 80 [60;80] | 80 [60;100] | 80 [60;80] | 100 [80;100] | 100 [80;100] | 100 [80;100] | 100 [80;100] | 100 [80;100] |
| **Periportal fibrosis** | Yes | 19 | 60 [60;80] | 80 [60;80] | 80 [60;80] | 100 [80;100] | 60 [40;80] | 80 [60;85] | 70 [60;80] | 100 [80;100] | 100 [75;100] | 100 [80;100] | 80 [80;100] | 90 [80;100] |
|  | No | 95 | 80 [60;80] | 80 [80;80] | 70 [60;80] | 100 [90;100] | 80 [60;80] | 80 [65;100] | 80 [60;80] | 100 [80;100] | 100 [80;100] | 100 [100;100] | 100 [80;100] | 100 [80;100] |
| **Liver steatosis** | Yes | 42 | 80 [60;80] | 80 [80;80] | 70 [60;80] | 100 [90;100] | 80 [55;80] | 80 [70;100] | **60 [60;80]*** | 100 [80;100] | 90 [75;100] | 100 [80;100] | 80 [80;100] | 100 [80;100] |
|  | No | 72 | 80 [60;80] | 80 [60;80] | 80 [60;90] | 100 [80;100] | 80 [60;80] | 80 [60;100] | **80 [60;80]** | 100 [80;100] | 100 [80;100] | 100 [85;100] | 100 [80;100] | 100 [80;100] |
| **Decreased  velocity of PV** | Yes | 11 | 80 [80;80] | 80 [80;80] | 80 [48;83] | 95 [60;100] | 80 [80;80] | 80 [60;100] | 80 [60;100] | 80 [60;100] | 100 [100;100] | 100 [80;100] | 80 [80;100] | 90 [80;100] |
|  | No | 103 | 80 [60;80] | 80 [60;80] | 70 [60;80] | 100 [80;100] | 60 [60;80] | 80 [70;100] | 80 [60;80] | 100 [80;100] | 100 [80;100] | 100 [90;80] | 100 [80;100] | 100 [80;100] |
| **Increased TE** | Yes | 7 | 80 [60;80] | 80 [60;100] | 80 [60;100] | 100 [90;100] | 80 [60;80] | 80 [80;100] | 80 [60;100] | 80 [80;100] | 100 [80;100] | 100 [100;100] | 80 [80;100] | 100 [80;100] |
|  | No | 107 | 80 [60;80] | 80 [80;80] | 70 [60;80] | 100 [80;100] | 80 [60;80] | 80 [60;100] | 80 [60;80] | 100 [80;100] | 100 [80;100] | 100 [100;100] | 100 [80;100] | 100 [80;100] |
| **Hepatomegaly** | Yes | 35 | 80 [60;80] | 80 [80;80] | 70 [60;80] | 100 [80;100] | 80 [80;100] | 80 [80;100] | 80 [60;80] | 100 [80;100] | 100 [80;100] | 100 [80;100] | 80 [80;100] | 100 [80;100] |
|  | No | 79 | 80 [60;80] | 80 [60;80] | 70 [60;80] | 100 [90;100] | 60 [40;80] | 80 [60;100] | 80 [60;80] | 100 [80;100] | 100 [80;100] | 100[ 100;100] | 100 [80;100] | 100 [80;100] |
| **Splenomegaly** | Yes | 12 | 80 [80;95] | 80 [60;80] | 80 [70;98] | 100 [90;100] | 80 [45;80] | 80 [65;80] | 80 [60;95] | 80 [80;100] | 80 [50;100] | 100 [100;100] | 80 [65;100] | 100 [80;100] |
|  | No | 102 | 80 [60;80] | 80 [80;80] | 70 [60;80] | 100 [80;100] | 80 [60;80] | 80 [60;100] | 80 [60;80] | 100 [80-100] | 100 [80-100] | 100 [80-100] | 100 [80-100] | 100 [80-100] |

|  | **CFAbd-Score** *- continued* | | | | | | | | | | | | | |
| --- | --- | --- | --- | --- | --- | --- | --- | --- | --- | --- | --- | --- | --- | --- |
|  | **DoEA-3** | | | **SSC-2** | | **GISrQoL-9** | | | | | | | | |
| **US-findings** | LoA | NFE | LoT | ASCn | ASCl | Emb | PAL | RP | Fat | RC | Fru | Sad | DFA | WUN |
| **Thickened  bowel wall** | 90 [60;100] | 100 [95;100] | 100 [80;100] | 100 [0;100] | 100 [100;100] | 80 [60;100] | 100 [60;100] | 80 [60;100] | 60 [40;100] | 70 [40;100] | 80 [40;100] | 100 [60;100] | 80 [60;100] | 80 [60;100] |
|  | 80 [60;100] | 100 [80;100] | 100 [80;100] | 100 [40;100] | 100 [100;100] | 100 [80;100] | 80 [60;100] | 100 [70;100] | 80 [60;100] | 80 [80;100] | 80 [60;100] | 100 [80;100] | 80 [60;100] | 80 [60;100] |
| **Enlarged  lymph nodes** | 80 [55;100] | 100 [55;100] | 100 [100;100] | 100 [40;100] | 100 [100;100] | 100 [75;100] | 100 [75;100] | 100 [90;100] | 100 [80;100] | 100 [80;100] | 90 [75;100] | 100 [75;100] | 100 [75;100] | 90 [60;100] |
|  | 80 [60;100] | 100 [80;100] | 100 [80;100] | 100 [40;100] | 100 [100;100] | 100 [80;100] | 80 [60;100] | 80 [60;100] | 80 [60;100] | 80 [60;100] | 80 [60;100] | 100 [80;100] | 80 [60;100] | 80 [60;100] |
| **Appendiceal thickening** | 80 [60;100] | 100 [80;100] | 100 [100;100] | 90 [30;100] | 100 [100;100] | 100 [100;100] | 100 [100;100] | 100 [80;100] | 100 [60;80] | 100 [80;100] | 100 [80;100] | 100 [80;100] | 100 [100;100] | 100 [60;100] |
|  | 80 [60;100] | 100 [80;100] | 100 [80;100] | 100 [40;100] | 100 [100;100] | 100 [80;100] | 80 [60;100] | 80 [60;100] | 80 [60;100] | 80 [60;100] | 80 [60;100] | 100 [80;100] | 80 [60;100] | 80 [60;100] |
| **Free fluid** | 100 [60;100] | 100 [80;100] | 100 [100;100] | 100 [40;100] | 100 [100;100] | 100 [60;100] | 100 [80;100] | 100 [80;100] | 100 [60;100] | 100 [60;100] | 80 [60;100] | 100 [80;100] | 100 [80;100] | 100 [60;100] |
|  | 80 [60;100] | 100 [80;100] | 100 [80;100] | 100 [40;100] | 100 [100;100] | 100 [80;100] | 80 [60;80] | 80 [60;100] | 80 [60;100] | 80 [60;100] | 80 [60;100] | 100 [80;100] | 80 [60;100] | 80 [60;100] |
| **Pancreatic cystosis** | 100 [75;100] | 100 [75;100] | 80 [55;100] | 90 [0;100] | 100 [100;100] | 80 [55;100] | 70 [35;85] | 70 [20;85] | 70 [60;100] | 80 [80;100] | 90 [55;100] | 100 [95;100] | 90 [75;100] | 80 [75;100] |
|  | 80 [60;100] | 100 [80;100] | 100 [80;100] | 100 [40;100] | 100 [100;100] | 100 [80;100] | 80 [80;100] | 100 [80;100] | 80 [60;100] | 80 [60;100] | 90 [60;100] | 100 [80;100] | 80 [60;100] | 80 [60;100] |
| **Pancreatic lipomatosis** | 80 [60;100] | 100 [80;100] | 100 [80;100] | 100 [70;100] | 100 [100;100] | 100 [80;100] | 80 [60;100] | 80 [60;100] | 80 [60;100] | 80 [60;100] | 80 [60;100] | 100 [80;100] | 80 [60;100] | 80 [60;100] |
|  | 80 [40;100] | 100 [50;100] | 100 [100;100] | 80 [40;100] | 100 [100;100] | 100 [80;100] | 100 [80;100] | 100 [80;100] | 100 [70;100] | 100 [80;100] | 100 [60;100] | 100 [100;100] | 100 [70;100] | 90 [80;100] |
| **Micro-gallbladder** | 80 [60;80] | 100 [60;80] | 100 [60;80] | 100 [60;80] | 100 [60;80] | 100 [60;80] | 80 [60;80] | 80 [60;80] | 80 [60;80] | 80 [60;80] | 80 [60;80] | 80 [60;80] | 80 [60;80] | 80 [60;80] |
|  | 80 [60;80] | 100 [60;80] | 100 [60;80] | 100 [60;80] | 100 [60;80] | 100 [60;80] | 80 [60;80] | 100 [60;80] | 80 [60;80] | 80 [60;80] | 90 [60;80] | 100 [60;80] | 100 [60;80] | 80 [60;80] |
| **Coarse/Irr. hep. parench.** | 100 [80;100] | 100 [100;100] | 100 [80;100] | 100 [10;100] | 100 [100;100] | 100 [80;100] | 80 [80;100] | 90 [80;100] | 80 [60;100] | 80 [60;100] | 80 [60;100] | 100 [70;100] | 100 [70;100] | 80 [60;100] |
|  | 80 [60;100] | 100 [80;100] | 100 [80;100] | 100 [40;100] | 100 [100;100] | 100 [60;100] | 80 [60;100] | 80 [60;100] | 80 [60;100] | 80 [60;100] | 80 [60;100] | 100 [80;100] | 80 [60;100] | 80 [60;100] |
| **Nodular  liver edge** | 100 [80;100] | 100 [100;100] | 100 [80;100] | 100 [75;100] | 100 [100;100] | 100 [100;100] | 100 [100;100] | 100 [80;100] | 100 [80;100] | 100 [80;100] | 100 [80;100] | 100 [80;100] | 100 [80;100] | 100 [80;100] |
|  | 80 [60;100] | 100 [80;100] | 100 [80;100] | 100 [40;100] | 100 [100;100] | 100 [80;100] | 80 [60;100] | 80 [60;100] | 80 [60;100] | 80 [60;100] | 80 [60;100] | 100 [80;100] | 80 [60;100] | 80 [60;100] |
| **Periportal fibrosis** | 80 [60;100] | 100 [80;100] | 100 [80;100] | 100 [0;100] | 100 [100;100] | 80 [75;100] | 80 [40;100] | 80 [40;100] | 80 [40;100] | 80 [60;100] | 80 [40;100] | 100 [75;100] | 80 [60;100] | 80 [60;100] |
|  | 80 [60;100] | 100 [80;100] | 100 [80;100] | 100 [40;100] | 100 [100;100] | 100 [80;100] | 80 [60;100] | 100 [80;100] | 80 [60;100] | 80 [60;100] | 80 [60;100] | 100 [80;100] | 80 [60;100] | 80 [60;100] |
| **Liver steatosis** | 90 [80;100] | 100 [100;100] | 100 [80;100] | 100 [20;100] | 100 [100;100] | 100 [60;100] | 80 [80;100] | 80 [75;100] | 80 [55;100] | 80 [60;100] | 80 [60;100] | 100 [75;100] | 100 [60;100] | 80 [60;100] |
|  | 80 [60;100] | 100 [65;100] | 100 [80;100] | 100 [40;100] | 100 [100;100] | 100 [80;100] | 80 [60;100] | 100 [60;100] | 80 [60;100] | 80 [60;100] | 80 [60;100] | 100 [80;100] | 80 [60;100] | 80 [80;100] |
| **Decreased**  **velocity of PV** | 80 [60;100] | 100 [60;100] | 100 [80;100] | 100 [40;100] | 100 [100;100] | 80 [60;100] | 80 [80;100] | 80 [80;100] | 80 [60;80] | 80 [80;100] | 80 [40;100] | 80 [60;100] | 80 [60;100] | 80 [60;80] |
|  | 80 [60;100] | 100 [60;100] | 100 [80;100] | 100 [40;100] | 100 [100;100] | 100 [80;100] | 80 [60;100] | 80 [60;100] | 80 [60;100] | 80 [60;100] | 80 [60;100] | 100 [80;100] | 80 [60;100] | 80 [60;100] |
| **Increased TE** | 80 [80;100] | 100 [100;100] | 100 [80;100] | 80 [0;100] | 100 [100;100] | 100 [100;100] | 80 [80;100] | 80 [80;100] | 80 [60;100] | 80 [60;100] | 80 [80;100] | 100 [80;100] | 100 [80;100] | 80 [80;100] |
|  | 80 [60;100] | 100 [80;100] | 100 [80;100] | 100 [40;100] | 100 [100;100] | 100 [80;100] | 80 [60;100] | 90 [60;100] | 80 [60;100] | 80 [60;100] | 80 [60;100] | 100 [80;100] | 80 [60;100] | 80 [60;100] |
| **Hepatomegaly** | 80 [60;100] | 100 [60;100] | 100 [80;100] | 100 [80;100] | 100 [100;100] | 80 [60;100] | 80 [80;100] | 80 [60;100] | 80 [60;100] | 80 [60;100] | 100 [60;100] | 100 [80;100] | 100 [60;100] | 80 [60;100] |
|  | 80 [60;100] | 100 [80;100] | 100 [80;100] | 100 [0;100] | 100 [100;100] | 100 [80;100] | 80 [60;100] | 90 [65;100] | 80 [60;100] | 80 [60;100] | 80 [60;100] | 100 [80;100] | 80 [60;100] | 80 [60;100] |
| **Splenomegaly** | 90 [80;100] | 100 [85;100] | 100 [80;100] | 100 [40;100] | 100 [100;100] | 90 [60;100] | 80 [65;100] | 90 [80;100] | 80 [45;95] | 80 [60;100] | 80 [65;95] | 90 [65;100] | 80 [65;100] | 80 [60;80] |
|  | 80 [60;100] | 100 [80;100] | 100 [80;100] | 100 [40;100] | 100 [100;100] | 100 [80;100] | 80 [60;100] | 80 [60;100] | 80 [60;100] | 80 [60;100] | 80 [60;100] | 100 [80;100] | 90 [60;100] | 80 [60;100] |

^§^Medians of symptoms scores ranged from 0 (higher rates of symptoms) to 100 (no symptoms); Median, lower (Q_1_) and upper (Q_3_) interquartiles of the score in relation to the respective US parameter are given as median [Q_1_;Q_3_].

*p<0.05 according to Mann-Whitney-U; significance levels were not corrected for multiple testing. †Intussusception (n=2) and cholecystolithiasis (n=3) were excluded from the analysis due to the low number of patients. PV=portal vein flow, TE=transient elastography, AP-4=abdominal pain domain, NAP-8=non abdominal pain domain, DoEA-3=disorders of eating and appetite domain, SCC-2=stool consistency and color domain, GISrQoL-9=gastrointestinal symptoms related quality of domain, APF=abdominal pain frequency, APD=abdominal pain duration, API=abdominal pain intensity, APB=abdominal pain by bowel movements, Fla=flatulence, AD=abdominal distension, FS=fatty stools, Cnst=Constipation, HB=heartburn, Vom=vomiting, Nau=Nausea, Ref=reflux of stomach content, LoA=loss of appetite, NFE=need for a forced eating, LoT=loss of taste, ASCn=altered stool consistency, ASCl=altered stool color, Emb=embarrassed, PAL=physical activity limitation, RP=reduced productivity, Fat=fatigue, RC=reduced concentration, Fru=Frustated, DFA=difficulty falling asleep, WUN=wake up at night

**Table S2: Relation of sonographic changes/pathology to quantity of supplemented pancreatic enzymes†**

|  | *Supplementation of enzymes  (units of lipase /kg of body weight/day)** | |  |
| --- | --- | --- | --- |
|  | B-US sign absent | B-US sign present | *p* value |
| Thickened bowel wall | 5084 [7813] | 4808 [5327] | .731 |
| Enlarged mesenteric lymph nodes | 4865 [6471] | 6673 [7054] | .275 |
| Appendiceal thickening | 4667 [6279] | 8048 [5281] | .132 |
| Free fluid in the abdominal cavity | 4667 [6544] | 7925 [6153] | .143 |
| Pancreatic cystosis | 4808 [6479] | 5457 [6158] | .967 |
| Pancreatic lipomatosis | 7143 [13757] | 4737 [6039] | .340 |
| Micro-gallbladder | 4520 [5823] | 6612 [7795] | .090 |
| Coarse/Irregular hepatic parenchyma | 4950 [7107] | 4808 [3519] | .819 |
| Nodular liver edge | 4922 [6058] | 5587 [9305] | .939 |
| Periportal fibrosis | 5245 [7040] | 3296 [4787] | .180 |
| Liver steatosis | 4936 [8593] | 4603 [4964] | .805 |
| Decreased velocity of the portal vein (PV) | 4922 [6912] | 6683 [6299] | .899 |
| Increased transient elastography (TE) | 4570 [6222] | 5597 [6335] | .702 |
| Hepatomegaly | 4950 [6279] | 4808 [7739] | .489 |
| Splenomegaly | 4936 [6887] | 5127 [3348] | .685 |

†Intussusception (n=2) and cholelithiasis (n=3) were excluded from the analysis due to the low number of patients

* Medians and interquartile ranges [IQR] are reported in the table

**Table S3:** Relation of sonographic changes/pathology to status of pancreatic sufficiency/insufficiency

|  | **PS** (n=8) | **PS🡪PI** (n=8) | **PI** (n=98) |
| --- | --- | --- | --- |
|  | n (%) | n (%) | n (%) |
| Thickened bowel wall | 0 (0%) | 0 (0%) | 26 (27%) |
| Enlarged mesenteric lymph nodes | 0 (0%) | 1 (13%) | 9 (9%) |
| Intussusception | 0 (0%) | 0 (0%) | 2 (2%) |
| Appendiceal thickening | 0 (0%) | 0 (0%) | 7 (7%) |
| Free fluid in the abdominal cavity | 0 (0%) | 0 (0%) | 11 (11%) |
| Pancreatic cystosis* | 1 (13%) | 0 (0%) | 5 (6%) |
| Pancreatic lipomatosis* | 6 (75%)† | 5 (63%) | 81 (91%) |
| Cholelithiasis | 1 (13%) | 0 (0%) | 2 (2%) |
| Micro-gallbladder | 1 (13%) | 1 (13%) | 22 (22%) |
| Coarse/Irregular hepatic parenchyma | 1 (13%) | 0 (0%) | 24 (25%) |
| Nodular liver edge | 1 (13%) | 0 (0%) | 6 (6%) |
| Periportal fibrosis | 1 (13%) | 0 (0%) | 18 (18%) |
| Liver steatosis | 1 (13%)† | 0 (0%) | 41 (42%) |
| Decreased velocity of the portal vein (PV) | 1 (13%) | 0 (0%) | 10 (11%) |
| Increased transient elastography (TE)** | 1 (13%) | 0 (0%) | 6 (7%) |
| Hepatomegaly | 1 (13%) | 2 (25%) | 32 (33%) |
| Splenomegaly | 1 (13%) | 1 (13%) | 10 (10%) |

*Pancreas was adequately visualized in 105/144 patients
†p <0.05 between PI and PS according to chi square test

**Transient elastography was evaluated in 87% of the patients (99/114)
PS = patients with pancreatic sufficiency; PI: patients with pancreatic insufficiency
PS**🡪**PI patients with primary PS who with time became PI
